# miRNAs expression signature potentially associated with lymphatic dissemination in locally advanced prostate cancer

**DOI:** 10.1186/s12920-020-00788-9

**Published:** 2020-09-18

**Authors:** Elena A. Pudova, George S. Krasnov, Kirill M. Nyushko, Anastasiya A. Kobelyatskaya, Maria V. Savvateeva, Andrey A. Poloznikov, Daniyar R. Dolotkazin, Kseniya M. Klimina, Zulfiya G. Guvatova, Sergey A. Simanovsky, Nataliya S. Gladysh, Artemy T. Tokarev, Nataliya V. Melnikova, Alexey A. Dmitriev, Boris Y. Alekseev, Andrey D. Kaprin, Marina V. Kiseleva, Anastasiya V. Snezhkina, Anna V. Kudryavtseva

**Affiliations:** 1 Engelhardt Institute of Molecular Biology, Russian Academy of Sciences, Moscow, Russia; 2 National Medical Research Radiological Center, Ministry of Health of the Russian Federation, Moscow, Russia; 3 Vavilov Institute of General Genetics, Russian Academy of Sciences, Moscow, Russia; 4 A. N. Severtsov Institute of Ecology and Evolution, Russian Academy of Sciences, Moscow, Russia; 5 MSAVM&B - MVA named after K.I. Skryabin, Moscow, Russia

**Keywords:** Prostate cancer, microRNA, Lymphatic dissemination, miRNA-Seq, Prognostic markers

## Abstract

**Background:**

Prostate cancer is one of the most common and socially significant cancers among men. The aim of our study was to reveal changes in miRNA expression profiles associated with lymphatic dissemination in prostate cancer and to identify the most prominent miRNAs as potential prognostic markers for future studies.

**Methods:**

High-throughput miRNA sequencing was performed for 44 prostate cancer specimens taken from Russian patients, with and without lymphatic dissemination (N1 – 20 samples; N0 – 24 samples).

**Results:**

We found at least 18 microRNAs with differential expression between N0 and N1 sample groups: *miR-182-5p, miR-183-5p, miR-96-5p, miR-25-3p, miR-93-5p, miR-7-5p, miR-615-3p, miR-10b, miR-1248* (N1-miRs; elevated expression in N1 cohort; *p* < 0.05); *miR-1271-5p, miR-184, miR-222-3p, miR-221-5p, miR-221-3p, miR-455-3p, miR-143-5p, miR-181c-3p* and *miR-455-5p* (N0-miRs; elevated expression in N0; *p* < 0.05)*.* The expression levels of N1-miRs were highly correlated between each other (the same is applied for N0-miRs) and the expression levels of N0-miRs and N1-miRs were anti-correlated. The tumor samples can be divided into two groups depending on the expression ratio between N0-miRs and N1-miRs.

**Conclusions:**

We found the miRNA expression signature associated with lymphatic dissemination, in particular on the Russian patient cohort. Many of these miRNAs are well-known players in either oncogenic transformation or tumor suppression. Further experimental studies with extended sampling are required to validate these results.

## Background

Prostate cancer (PC) is the second most common cause of death from cancer among men worldwide [1]. The issue of making an informed decision on PC treatment options after radical prostatectomy (with expanded pelvic lymphadenectomy) remains open. This choice is usually based on the stratification of patients into risk groups depending on several parameters: tumor stage, Gleason index, PSA level, and regional metastasis. Nevertheless, these clinical indicators are not enough, and selecting for a patient group with high potential for tumor progression and further development of aggressive phenotype is one of the most important unsolved tasks of clinical oncology [2, 3]. Therefore, additional reliable markers are needed to guide decisions about treatment type [4].

One promising PC-specific prognostic marker is expression of the fusion transcript TMPRSS2-ERG. The chimeric TMPRSS2-ERG gene is the fusion of the androgen-regulated TMPRSS2 gene with ERG, the most representative member of the oncogenic ETS transcription factor family. It has been demonstrated that TMPRSS2-ERG is found in 40% of cases of prostate adenocarcinoma [5]. Expression level of TMPRSS2-ERG in the urine negatively correlates with the degree of tumor differentiation (according to Gleason score), and this profile can be used for risk stratification of PC patients [6].

Currently, several commercially available kits allow risk assessment of both tumor progression and aggressive tumor phenotype development (e.g. Oncotype DX Prostate Cancer Assay, Prolaris Score). These commercial kits attempt to predict prognosis after radical prostatectomy; however, none of the available prognostic systems predicts lymphatic dissemination [7].

According to numerous studies, the search for biomarkers is focused on microRNAs (miRNAs) [8, 9]. miRNAs are involved in various cellular pathways, such as proliferation and differentiation, by affecting the expression of their target genes [10, 11].

The miRNA expression profiles of normal and tumor tissues are noticeably different, which can be used to discriminate these tissues [12]. Notably, miRNA profiling seems to have better accuracy in differentiating tumor from normal tissue compared to mRNA profiling [13]. miRNA expression profiles also frequently correspond to clinical and pathological parameters, and thus can predict the patient’s response to therapy [14, 15]. This emphasizes the potential of miRNAs as prognostic markers.

In this study, we performed miRNA-Seq of tumor tissues from Russian patients diagnosed with locally advanced PC (LAPC). Our goal was to identify miRNA expression signatures associated with lymphatic dissemination and to clarify the role of these miRNAs in the development of cell metastatic potential.

## Methods

### Tumor tissue samples

PC tissues samples were obtained from patients who underwent surgical intervention in the P.A. Hertzen Moscow Oncology Research Center (branch of the National Medical Research Radiological Center, Ministry of Health of the Russian Federation) in 2015–2016. All material was collected and characterized by the organization’s Pathology Department according to the WHO Classification of Tumors of the Urinary System and Male Genital Organs [16]. Each sample contained a minimum of 70% tumor cells. Tissue samples were immediately frozen and stored in liquid nitrogen following surgical resection.

In the current study, we used 44 LAPC (adenocarcinoma) samples with negative (*n* = 24) and positive lymph nodes (*n* = 20) obtained from patients who underwent surgical treatment and did not receive neoadjuvant therapy. Samples have the following characteristics: pT3a and pT3b categories, negative resection margin, any PSA value, and any Gleason score (Table 1). We did not include normal prostate tissues in the current study.

**Table 1:** Clinicopathological characteristics of Russian patients

	Patients N0	Patients N1
Age (mean)	51–73 (64)	57–73 (64)
PSA (ng/ml)	4–27,6	7,4-18,41
Pathological stage (T)	T3a/3b	T3a/3b
Pathological Gleason	6–9	7–9
Surgical margin	negative	negative
Neoadjuvant therapy	no	no

The study was approved by The Ethics committee of P.A. Hertzen Moscow Oncology Research Center, Ministry of Health of the Russian Federation. The study was done in accordance with the principles outlined in the Declaration of Helsinki (1964). All patients provided written informed consent.

### RNA isolation, library preparation, and next generation sequencing (NGS)

Total RNA was isolated from 44 tumor tissue samples using a MagNA Pure Compact RNA Isolation Kit (Roche, Switzerland). RNA concentration was determined using a Qubit 2.0 Fluorometer (Thermo Fisher Scientific, USA). The RNA integrity number (RIN) was evaluated using an Agilent 2100 Bioanalyzer (Agilent Technologies, USA). RIN for all samples studied was no less than 7.0, on a scale of 1–10.

Libraries were prepared using an NEBNext® Small RNA Library Prep Set for Illumina (New England Biolabs, USA) according to the manufacturer’s protocol. Libraries were validated prior to sequencing using quantitative PCR (qPCR). Sequencing was performed on a NextSeq500 System (Illumina) at the EIMB RAS “Genome” Center [http://www.eimb.ru/rus/ckp/ccu_genome_c.php].

### miRNA-Seq data analysis

To process miRNA-Seq data, we used the miRge 2.0 pipeline [17]. Besides de novo miRNA prediction, this pipeline provides annotation of miRNA-seq data against a known miRNA database. In brief, miRge 2.0 trims adapter sequences with the Cutadapt package, maps reads to miRBase v22 [18]) as well as tRNA, snoRNA, ncRNA and mRNA datasets, and calculates miRNA abundance. Next, we analyzed read counts data using the edgeR package [19] and compared N0 and N1 groups. We used TMM normalization and then calculated CPMs considering normalization factors derived with TMM method (e.g. CPM = read_counts_per_miRNA / total_read_count * 1.000.000 / TMM_norm_factor). Additionally, we compared several normalization methods: RLE, TMM, upper quartile and ‘total read count’. Next, we applied quasi-likelihood F-test (QLF) and the Mann-Whitney (MW) non-parametric test. Benjamini-Hochberg adjustment was applied to calculate the false discovery rate (FDR).

miRNA expression level differences were treated as statistically significant if they underwent a 1.5-fold change or greater and for both QLF and MW tests had FDR < 0.05. Additionally, for each miRNA, we calculated portions of samples with high and low relative expression (lower than average/1.5 or higher than average*1.5) and compared these frequencies between N0 and N1 cohorts.

Next, we tried to identify the pathways most affected by differentially expressed (DE) miRNAs. For this purpose, we used the mirPath 3.0 (DIANA tools) web service [20] and TarBase 7.0 data [21]. Visualization of the number of miRNAs targeting members of KEGG pathways was performed with the pathview package (R, Bioconductor) [22]. Additionally, we performed association network reconstruction and GO, KEGG, Reactome, SMART, InterPro, and Pfam enrichment using the STRING web service [23].

## Results and discussion

### MicroRNAs associated with lymphatic dissemination

A total of 301 miRNAs passed the read-counts-per-million (CPM) thresholds for further analysis (Additional file 1). Among these, 18 miRNAs passed the statistical significance threshold *p* < 0.05 (both Mann-Whitney (MW) and quasi-likelihood F-test (QLF) tests), and 1.5-fold expression level changes between N0 and N1 groups. Among these 18 microRNAs, there are 9 ones that are overexpressed in N1 tumors, compared to N0 (further we will denote these microRNAs as “N1-miRs”): *miR-182-5p, miR-183-5p, miR-96-5p, miR-25-3p, miR-93-5p, miR-7-5p, miR-615-3p, miR-10b and miR-1248*; and 9 miRs that are underexpressed in N1 tumors, compared to N0 (further – N0-miRs): *miR-1271-5p, miR-184, miR-222-3p, miR-221-5p, miR-221-3p, miR-455-3p, miR-143-5p, miR-181c-3p and miR-455-5p*.

**Fig. 1:**
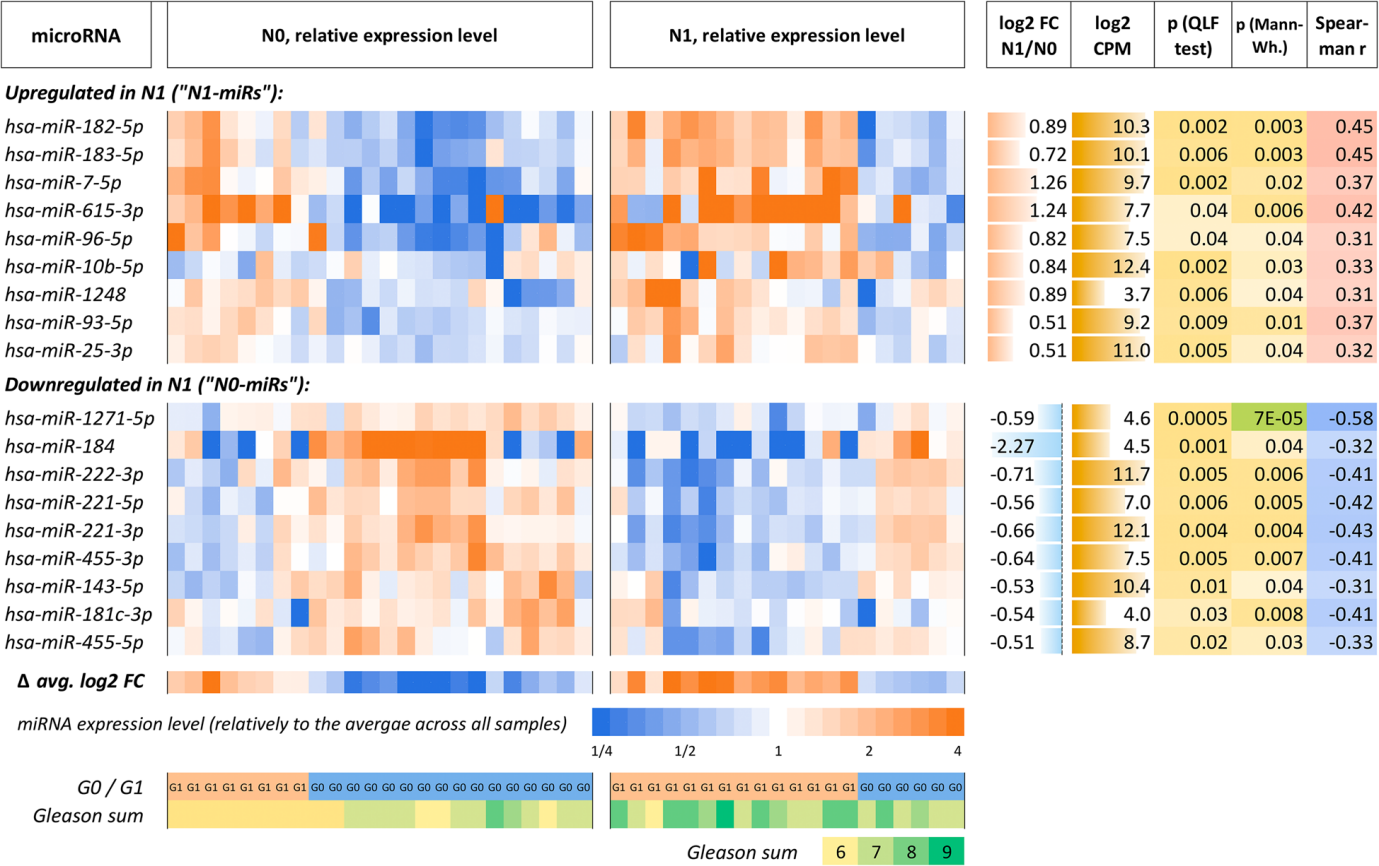
Top microRNAs that discriminate N0 and N1 groups (*p* < 0.05, both QLF and MW). log2 FC – binary logarithm of expression level ratio between N1 and N0; log2 CPM – binary logarithm of read counts per million (CPM); p (QLF test) – p-value according to quasi-likelihood F-test (edgeR); p (Mann-Wh.) – *p*-value according to Mann-Whitney U test; Spearman r – Spearman’s rank correlation coefficient between miRNA expression level and N0/N1 feature; Δ avg. log2 FC – difference of average log2 FC between N1-miRs and N0-miRs. The samples are divided into two subgroups: G0 (high expression of N0-miRs and low expression of N1-miRs; associated with N0) and G1 (low expression of N0-miRs and high expression of N1-miRs; associated with N1)

Notably, all the nine N1-miRs are highly correlated to each other, with except for miR-10b-5p. The average pairwise Spearman’s rank correlation coefficient is *r* = 0.63; for almost all pairs, *p* < 0.001 (Additional file 2). Similarly, all nine N0-miRs are also highly self-correlated: average *r* = 0.54, and for the most of pairs, *p* < 0.001. In turn, all N1-miRs are anti-correlated to N0-miRs: average *r* = − 0.55, and *p* < 0.001 for the most of pairs, except for miR-10b-5p. In other words, we can speak about a microRNA expression signature characteristic of tumors with lymphatic dissemination. On the Fig. 1, the difference of average *log2 FC* values between N1-miRs and N0-miRs is designated as *Δ avg. log2 FC*. Hence, we see that the samples are divided into two groups: G0 (with *Δ avg. log2 FC* < 0) and G1 (with *Δ avg. log2 FC* > 0). N1 cohort is significantly enriched with G1 samples, and N0 cohort – with G0 samples (*p* = 0.03, Fisher’s exact test).

Additionally, we performed comparison of several data normalization methods (TMM, RLE, upper quartile, total read count). These methods gave almost identical results; ‘total read count’ normalization method mostly diverged from other ones.

It is expected that the degree of cancer cell differentiation should be negatively associated with lymphatic dissemination, and contribute to the increased invasion. In other words, Gleason score, which is negatively associated with cell differentiation degree, should correlate with the presence of lymph node metastases. Indeed, Gleason score strongly correlated with N0-N1 (Spearman’s *r* = 0.45, *p* = 0.002). Moreover, expression levels of several N1-miRs (miR-182-5p, miR-183-5p, miR-615-3p, miR-25-3p) positively correlated with Gleason score (*r* = 0.32…0.45, *p* < 0.05), and expression of the most of N0-miRs (miR-184, miR-222-3p, miR-221-3p/5p, miR-143-5p, miR-455-5p) was negatively associated (*r* = − 0.32…-0.44, *p* < 0.05).

### Upregulated microRNAs associated with lymphatic dissemination (N1-miRs)

According to the results of microRNAs expression profiling of 48 LAPC samples, the following microRNAs upregulated in N1 (compared to N0) have been identified: *miR-182-5p, miR-183-5p, miR-96-5p, miR-25-3p, miR-93-5p, miR-7-5p, miR-615-3p, miR-10b and miR-1248* (denoted as N1-miRs).

Three of the identified microRNAs, miR-182-5p, − 183-5p and − 96-5p, are members of the miR-183-96-182 oncogenic cluster. Various studies demonstrate that the miR-183-96-182 cluster plays an important role in oncogenesis, cancer progression, tumor invasion and metastasis. Thus, miR-183 supports tumor invasion and metastasis by targeting target genes such as *PDCD4, PP2A, EGR1*, and *PTEN* [24–26]. The well-known prostate tumor suppressor *PTEN* has been shown to be regulated by miR-183-5p and miR-96-5p and promote cell proliferation in breast cancer by directly affecting the tumor suppressor gene *FOXO3a* and cyclin-dependent kinase inhibitors p27^Kip1^ and p21^Cip1^ [27].

All three miRs, (96, 182 and 183) cause downregulation of *FOXO1* tumor suppressor, which induce cell cycle arrest and cell death in endometrial cancer [28]. Additionally, *FOXO1* has been suggested to act as a repressor of androgen receptor activity, which is the central oncogenic pathway in the development of prostate cancer [29]. There is a report that metastasis of prostate cancer is promoted by stimulation of the *TGF-β* and *SMAD* genes by inducing miR-96 and activating the mTOR pathway [30]. The *TGF-β* gene also activates miR-182, which can affect the *CYLD* gene, which can further contribute to the activation of NF-κB, which was demonstrated in the case of glioblastoma, and subsequently lead to angiogenesis and tumor invasion [31]. Increased expression of miR-182-5p was demonstrated in prostate cancer tissues compared with normal tissue, and moreover, the expression level of miR-182-5p allows us to distinguish tumor from non-tumor tissue with 100% specificity [32–34]. Thus, miR-183-96-182 cluster is prospective prostate cancer prognosis biomarker candidate, and further studies on an extended cohort are needed to accurately assess its clinical significance and the limits of applicability.

Two other microRNAs with predominantly increased expression level in N1 samples, miR-25 and miR-93, along with miR-106 are the members of a highly conserved oncogenic miR-106b-25 cluster. It is overexpressed in many types of cancer, including gastric, prostate, and pancreatic neuroendocrine tumors, neuroblastoma and multiple myeloma [35]. The miR-106b-25 clusters, as well as the miR-17-92 oncogenic cluster, are key modulators of transforming growth factor beta (TGF-β) signaling. Inactivation of TGF-β tumor pathway is a major step in the development of various human tumors. Modulation of TGF-β signaling in tumors thus prevents cell cycle arrest and apoptosis when overexpressed in cancer cells [35]. Previous studies have reported that miR-25-3p is associated with various types of tumors, including prostate cancer [36]. MiR-25 has been identified as an important regulator of invasive programs in non-transformed and malignant epithelial tissues of the human prostate gland. It was previously reported that in primary tumors and distant metastases in prostate cancer, increased regulation of miR-25-3p is observed [37, 38]. miR-93-5p likewise has an association with various types of cancer [39, 40]. Increased expression of exosomal miR-93-5p in plasma is significantly associated with both the risk of esophageal cancer and a poor prognosis for the disease [39]. In prostate cancer, increased expression of miR-93-5p associated with progression has been described [41].

Regarding miR-7-5p, it has been shown that it acts as a tumor suppressor in certain types of human cancers [42–44]. However, the role of miR-7-5p in prostate cancer remains to be explored. Aberrant expression of miR-615-3p has been described in many cancers, including prostate cancer, where increased expression of miR-615-3p has been observed in the most aggressive forms [45–47]. Experiments on cell lines of various types of cancer have shown that overexpression of miR-615-3p supports cell proliferation and migration [45, 46]. Functional studies carried out on PC3M prostate cancer cell lines showed that miR-615-3p promotes the proliferation, apoptosis and migration of PC3M prostate cancer cells in vitro, which indicates that miR-615-3p is a significant oncogenic microRNA in prostate cancer [47].

The presence of miR-10b was detected in various types of malignant neoplasms [48, 49]. It has been shown that increased expression of miR-10b was observed in metastatic melanoma cells and was positively associated with lymph node metastasis, a progressive clinical stage, and poorer survival [48]. A similar association of increased expression of miR-10b with lymph node metastases has been described in gastric cancer [50]. The least miRNA upregulated in N1, miR-1248, is poorly studied, and so far no reports have addressed to its biological function.

### Downregulated microRNAs associated with lymphatic dissemination (N0-miRs)

Among microRNAs with lowered expression level in N1 samples compared to N0, the following ones have passed the statistical significance thresholds: *miR-1271-5p, miR-184, miR-222-3p, miR-221-5p, miR-221-3p, miR-455-3p, miR-143-5p, miR-181c-3p and miR-455-5p* (denoted as N0-miRs).

Among them, there are several tumor suppressors microRNAs, but the role of the most of them may be dual and depends on a current biological context. MiR-1271-5p acts as a tumor suppressor in breast cancer, inhibits cell proliferation by suppressing *SPIN1* gene [51]. In the case of hepatocellular carcinoma, a decreased expression of miR-1271-5p is also observed, which may be associated with oncogenic effects [52]. In the case of osteosarcoma, it was demonstrated that miR-1271-5p inhibited cell proliferation and invasion by Wnt signaling [53]. A study in cell cultures reports inhibition of miR-1271-5p expression in ovarian cancer cells compared to normal [54].

Reduced expression of miR-184 was also detected in various types of human cancer, including renal cell [55], nasopharyngeal cancer [56], neuroblastoma [57], non-small cell lung cancer [58], glioma and breast cancer [59]. MiR-184 has been shown to act as tumor suppressor, and downregulated miR-184 level may be a predictor of poor prognosis in patients with non-small cell lung cancer [58].

Two other microRNAs that are decreased in N1 cohort, MiR-221 and miR-222, represent two partially homologous miRNAs that are encoded in tandem on chromosome X. In prostate cancer cells PC3, they target cell cycle inhibitor p27^Kip1^ and thus affect PC3 proliferation potential [60]. Normally, these miRs mediate vascular remodeling, a response to vascular injury, by controlling endothelial cells differentiation and inhibiting their proliferation and migration [61, 62]. These miRNAs have been extensively studied in many human malignancies (including prostate cancer), and their act either as tumor suppressors or oncogenes [60, 63–65]. It was revealed that miR-221 (both 3p and 5p) are downregulated during prostate cancer progression [66], miR-221-5p acts as tumor suppressor miRNA in prostate cancer cell lines and reduces tumor burden in mouse and zebrafish in vivo models. miR-221 was identified as key regulator of a network of other miRNAs in prostate cancer and has the potential to drastically modulate cell physiology [67]. Thus, the biological role and underlying mechanisms of miR-221 and miR-222 in the pathogenesis of androgen-independent prostate cancer are still unclear.

Another microRNA with decreased expression level in N1, miR-455-3p is involved in many pathological processes. In comparison with normal tissues, miR-455-3p has been shown to have reduced expression in various cancer tissues [68, 69]. It was found that miR-455-3p expression in prostate cancer tissues was significantly reduced compared to normal, demonstrating that miR-455-3p is associated with prostate carcinogenesis. Also, experiments on cell cultures showed that increased expression of miR-455-3p inhibits the growth of prostate cancer cells in vitro and in vivo. These research results show that miR-455-3p plays the role of a tumor suppressor in the prostate cancer progression [70].

MiR-143-5p is downregulated in gastric cancer and gallbladder cancer tissues and that forced miR-143-5p expression suppresses cancer cell malignancy through targeting *COX-2* and *HIF1-α*, respectively [71, 72]. In the case of cervical cancer, miR-143-5p has been shown to reduce the expression level of the Cyclin D1 and Bcl-2 protein in vivo and in vitro. miR-143-5p inhibits the progression, proliferation, migration and invasion of the cervical cell cycle, and also inhibits apoptosis of cervical cancer cells, suppressing the expression levels of the ELK1, p-ELK1, C-fos, Cyclin D1 and Bcl-2 protein [73].

miR-181c is also involved in various pathological pathways, including the important role in various cancers, such as colorectal cancer, breast cancer, and stomach cancer [74–76]. In a study of osteosarcoma, it was found that a decrease in miR-181c expression level was associated with a poor prognosis and malignant clinical and pathological features. As a result of functional studies, it was shown that activation of miR-181c dramatically inhibited the proliferation, invasion and migration of osteosarcoma cells [77]. It was also revealed that the *SMAD7* gene was a direct functional target for miR-181c in osteosarcoma cells and that overexpression of miR-181c suppresses the signaling pathway of EMT and TGF-β in osteosarcoma cells through regulation of *SMAD7* [77]. Significant downregulation of miR-181c was also detected in the case of hepatocellular carcinoma when compared with normal tissues. miR-181c regulated migration, invasion, apoptosis, and proliferation of hepatocellular carcinoma cell lines in vitro and tumor development in vivo [78].

miR-455-5p is involved in many pathological processes, such as cell proliferation, apoptosis, migration, and invasion. miR-455-5p successfully suppresses cell viability and induces cell apoptosis by targeting *RAF1* gene in colorectal cancer [79] and miR-455-5p is significantly downregulated in gastric cancer cells and can inhibit the proliferation and invasion of human gastric cancer cells, as well as promote cell apoptosis by affecting *RAB18* gene [80]. In addition, low miR-455 expression was associated with multiple tumor nodes and progressive stage metastatic tumor nodes in hepatocellular cancer and could significantly inhibit the migration and invasion [81]. In case of prostate cancer, the miR-455-5p tumor suppressor function has been shown to significantly suppress proliferation and initiate apoptosis of prostate cancer cells. The authors also experimentally determined a functional target for miR-455-5p - *CCR5* gene [82].

### Pathway enrichment analysis of microRNAs over- and underexpressed in N1 group

Using DIANA mirPath v.3 web server and TarBase 7.0 data, we performed pathway enrichment analysis of nine N1-miRs and nine N0-miRs, which have passed statistical significance thresholds. For N1-miRs, we identified 66 enriched KEGG pathways (*p* < 0.05), 23 of which have passed *p* < 10^− 5^ threshold. Among them, we should mention the following ones: protein processing in endoplasmic reticulum (hsa04141), proteoglycans in cancer (hsa05205), cell cycle (hsa04110), adherens junction (hsa04520), FoxO signaling pathway (hsa04068), p53 signaling pathway (hsa04115), pathways in cancer (hsa05200), prostate cancer (hsa05215).

For N0-miRs, mirPath has found 33 pathways with *p <* 0.05, only 5 of which have passed *p <* 10^− 5^ threshold. Among these pathways, we noticed: protein processing in endoplasmic reticulum (hsa04141), mRNA surveillance pathway (hsa03015), proteoglycans in cancer (hsa05205), adherens junction (hsa04520), ECM-receptor interaction (hsa04512), Hippo signaling pathway (hsa04390), transcriptional misregulation in cancer (hsa05202), cell cycle (hsa04110). Surprisingly, several pathways take first places among the enriched pathways for both N1- and N0-miRs: protein processing in endoplasmic reticulum pathway, adherens junctions, proteoglycans in cancer, and cell cycle.

**Fig. 2:**
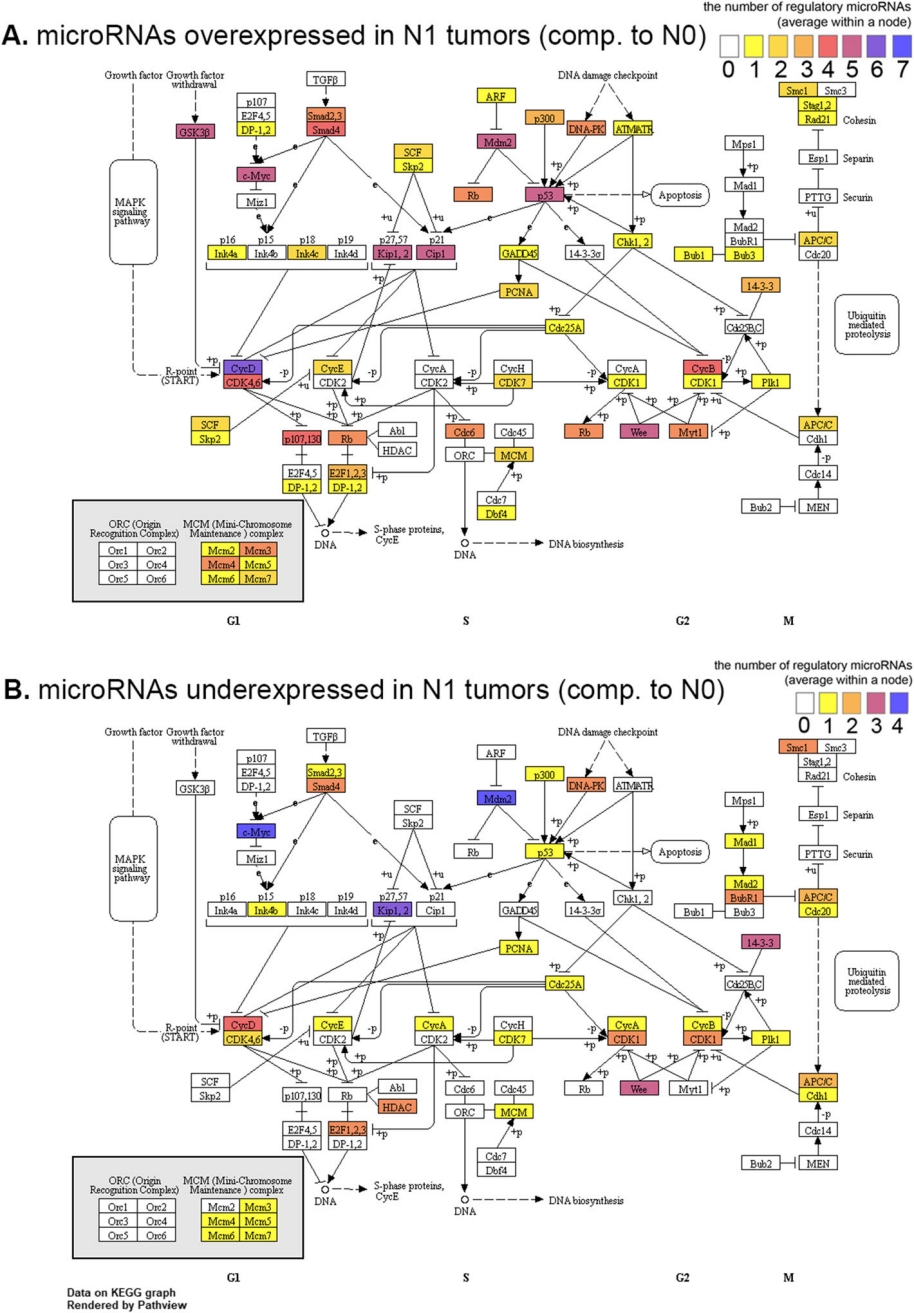
Targets of overexpressed (**a**) and downregulated (**b**) microRNAs (N1 versus N0) among genes participating cell cycle progression and regulation (KEGG pathway hsa04110 – cell cycle). Each rectangle corresponds to a single KEGG node, which can be represented either by one or several genes. The numbers of microRNAs between the genes comprising a single KEGG node are averaged (e.g. value for “Smad2,3” node is averaged between two genes, *SMAD2* and *SMAD3*)

Figure 2 demonstrates the number of targets of up- and down- regulated microRNAs among genes involved in cell cycle regulation, according to TarBase 7.0 data. Among them, N1-miRs (Fig. 2a) have more putative targets than N0-miRs (Fig. 2b), but the spectra of these targets are quite different. Among putative targets of N1-miRs (Fig. 2a), we noticed *SMAD2, SMAD3, SMAD4, p53, Rb, p27*^*Kip1*^*, p21*^*Cip1*^*, GADD45, Wee1, GSK3-β* which have tumor suppression activity and potential to inhibit cell cycle progression. Although some of these genes are also targeted by N0-miRs (for example p27^Kip1^), the overall number of mRNA targets with tumor suppression potential is greater for N1-miRs.

In general, we can say that N1-miRs demonstrate better coherence in the lists of mRNA targets rather than N0-miRs. The main reason is that the list of N1-miRs include a cluster of three microRNAs (miR-96/182/183). For example, there are 157 genes that are targeted by simultaneously 5 or more N1-miRs and only 11 genes are found as targets of simultaneously 5 or more N0-miRs (TarBase 7.0 data).

Besides this, we noticed a significant number of pro-oncogenes, which are targets of N0-miRs, whereby they may be suppressed in non-metastatic tumors:

- At least four N0-miRs (miR-181c-3p, 221-5p, 222-3p, 455-3p) target *c-Myc*, well known pro-tumorigenic transcription factor. c-Myc contributes to disease initiation and progression by stimulating an embryonic stem cell-like signature characterized by an enrichment of genes involved in ribosome biogenesis and by repressing differentiation [83]. In turn, the loss of cell differentiation is known to be associated with increases metastatic capability and disease recurrence [84].
- Several N0-miRs (both miR-221-3p/5p, both miR-455-3p/5p) target *THBS1* (thrombospondin 1) encoding for an adhesive glycoprotein that mediates cell-to-cell and cell-to-matrix interactions. It has negative impact on angiogenesis. This protein can bind to fibrinogen, fibronectin, laminin, type V collagen and integrins alpha-V/beta-1 and affects signal transduction. *THBS1* is multifaceted player in tumor progression; it affects tumor cell adhesion, invasion, migration, proliferation, apoptosis and tumor immunity [85]. The precise role of *THBS1* in tumor invasion and migration remains controversial, with compelling evidence suggesting both stimulatory and inhibitory roles [85].
- At least four microRNAs (miR-181c-3p, 221-3p, 222-3p, 1271-5p) target *MDM2*, an important negative regulator of the p53 tumor suppressor. MDM2 protein functions both as an E3 ubiquitin ligase that recognizes the N-terminal trans-activation domain (TAD) of the p53 tumor suppressor and as an inhibitor of p53 transcriptional activation. This is well known pro-oncogenic protein which represents a target for anti-tumor therapy (in p53-dependent manner), including prostate cancer [86–88].
- Several microRNAs also target *FRS2* (fibroblast growth factor receptor substrate 2), which participate signal transduction from receptor tyrosine kinases (RTKs) to multiple downstream signaling pathways, most notably the MAPK/ERK, PI3K/AKT/mTOR and PLCγ. It is overexpressed and amplified in several cancer types, including prostate cancer [89, 90].

Beyond them, we can notice several cancer-associated genes, which are putative targets of N0-miRs: *CCND1*, *CDK4/6*, laminin, syndecan, beta-catenin, *CREB*, *CCND2*, *MLL*. On other hand, we also see that N1-miRs target several gene with pro-oncogenic potential (member of Ras and ERK families, HIF1A and other ones).

**Fig. 3:**
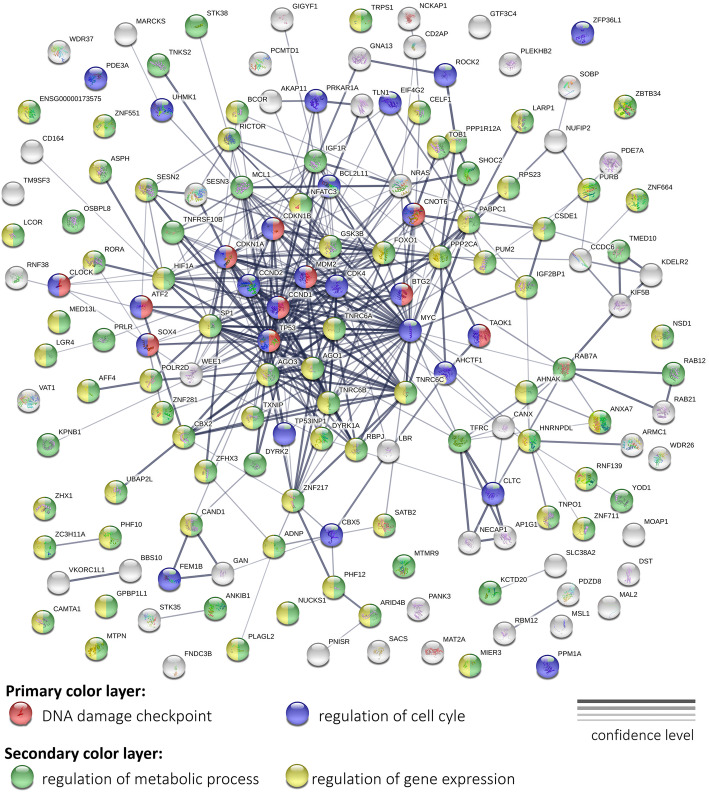
The interaction network of 157 genes that are targets of at least 5 microRNAs overexpressed in N1 tumors (N1-miRs). Foreground color layer indicates whether a gene participate regulation of the cell cycle (blue) and/or DNA damage checkpoints system (red). If a gene does not belong to these categories, but is involved in the regulation of cellular metabolism or the gene expression, then it is marked with yellow or green (background color layer)

Next, we selected 157 genes, which are targeted by at least 5 of our 9 N1-miRs, according to TarBase 7.0 data. For these genes, we performed network analysis and pathway enrichment using the STRING database (Fig. 3). The network that emerged is highly enriched with protein-protein interactions (*p <* 10^− 16^). This indicates that the genes and proteins we examined are biologically connected. As can be seen from the Fig. 3, network core consists of participants of cell cycle regulation, checkpoints, including *TP53, CCND1, CCND2, CDKN1A, BTG2, TAOK1, MYC, CNOT6* and other genes (marked with blue or red).

In addition, this list of 157 genes is highly enriched with participants of regulation of metabolic processes (GO:0019222), regulation of gene expression (GO:0010468), regulation of translation (GO:0006417), negative regulation of cell cycle (GO:0045786), mitotic DNA damage checkpoint (GO:0044773), gene silencing by miRNA (GO:0035195) and other terms (Additional file 3). Besides these GO terms, we observed enrichment with participants of Reactome pathways: Oncogene Induced Senescence (HSA-2559585), Transcriptional Regulation by TP53 (HSA-3700989); KEGG pathways: p53 signaling pathway (hsa04115), MicroRNAs in cancer (hsa05206), Cellular senescence (hsa04218), Prostate cancer (hsa05215). As can be seen, among them, most of the pathways in one way or another are associated with tumor suppression, as well as regulation of gene expression due to miRNAs. This is mainly about genes: *CCND1, CCND2, CDK4, CDKN1A, SESN2, SESN3, TNFRSF10B, TNRC6A/B/C, BTG2, TP53, PPP2CA*, most of them are tumor suppressors [91–94]. Of course, the list of enriched pathways was not limited to those associated with tumor suppression. Among them were the single pathways associated with tumor progression, for example, PI3K-Akt signaling pathway (Additional file 3).

Among protein domains encoded by 157 genes, we observed enrichment in Argonaute hook (*TNRC6A, TNRC6B, TNRC6C*), protein kinase domains (*CDK4, DYRK1A, DYRK2, GSK3B, IGF1R, ROCK2, STK35, STK38, TAOK1, UHMK1, WEE1*), RNA recognition motifs (*CELF1, HNRNPDL, IGF2BP1, PABPC1, RBM12, TNRC6C, UHMK1*), Argonaute domains (*AGO1, AGO3*). For the complete results of enrichment analysis, see the Supplementary Table S3. Thus, among potential targets of miRNAs, we see not only tumor suppressors, but also directly genes involved in miRNA-mediated suppression of expression, e.g. Argonaute proteins *AGO1/3* and Argonaute-navigating proteins *TNRC6A/B/C*.

**Fig. 4:**
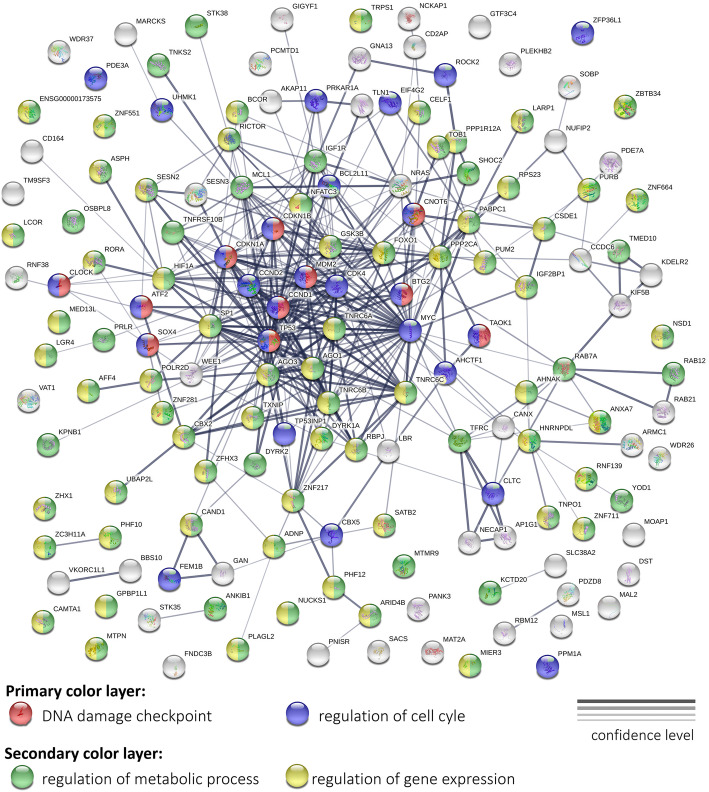
The interaction network of 139 genes that are targets of at least 3 microRNAs underexpressed in N0 tumors (N0-miRs). Foreground color layer indicates whether a gene participate negative regulation of apoptotic process (orange) and/or negative regulation of cell differentiation (blue). If a gene does not belong to these categories, but is involved in the regulation of cellular metabolism or the gene expression, then it is marked with yellow or green (background color layer)

Next, we performed the same procedure for 139 genes, the targets of at least 3 out of 9 N0-miRs (Fig. 4). The network is also highly enriched with protein-protein interactions (*p* = 7.5∙10^− 14^). Here, pro-oncogenic c-Myc is located in the center of the network and is connects to almost 30 peripheral nodes. Gene Ontology, KEGG and Reactome enrichment analyses demonstrated that the list of 139 genes contains elevated number of participants gene expression regulation (GO:0010468), regulation of metabolic process (GO:0019222), developmental process (GO:0032502), gene silencing by miRNA (GO:0035195), regulation of cell differentiation (GO:0045596; predominantly negative, GO:0045596), regulation of apoptotic process (GO:0042981; predominantly negative, GO:0043066); microRNAs in cancer KEGG pathway (hsa05206), PI3K-Akt signaling pathway (hsa04151); Transcriptional Regulation by TP53 (HSA-3700989). For the complete list of enriched pathways see the Additional file 4.

## Conclusions

We revealed at least 18 microRNAs with statistically significant difference in the expression profiles between N0 and N1 tumors: 9 N1-miRs, which are upregulated in lymph node-positive samples, and 9 N0-miRs, which are dowregulated in lymph node-positive samples. The expression levels of these microRNAs are significantly (anti)correlated to each other.

In turn, the samples examined can be divided into two groups (G1 and G0) according to the difference of expression levels between N0-miRs and N1-miRs. Lymph node positive (N1) group is significantly enriched with G1 tumors.

Many N1-miRs serve as putative negative regulators of genes participating cell cycle arrest and inhibiting tumor growth, including several tumor suppressors *CCND1, CCND2, CDK4, CDKN1A, SESN2, SESN3, TNFRSF10B, TNRC6A/B/C, BTG2, TP53, PPP2CA*. In turn, many N0-miRs can serve as putative suppressors of genes participating cell cycle progression, negative regulation of cell differentiation.

## Supplementary information

**Additional file 1 Table S1**. Total of 301 miRNAs passed the read-counts-per-million (CPM) thresholds.

**Additional file 2 Table S2.** Spearman’s correlation analysis of microRNAs associated with lymphatic dissemination. (

**Additional file 3 Table S3.** The enriched pathways list of 157 genes that are targets of at least 5 microRNAs overexpressed in N1 tumors.

**Additional file 4 Table S4.** The enriched pathways list of 139 genes that are targets of at least 3 out of 9 microRNAs overexpressed in N0 tumors.

## Data Availability

All data generated or analyzed in this study are included in the published article. microRNA transcriptome sequence data are available at the NCBI Sequence Read Archive (BioProject PRJNA562642).

## Abbreviations

PC: Prostate cancer
LAPC: Locally advanced prostate cancer
CPM: Read counts per million
LogCPM: Binary logarithm of reads counts per million
QLF test: Quasi-likelihood F-test
Mann-Wh.: Mann-Whitney U-test
LogFC: Binary logarithm of expression level fold change
Spearman: Spearman’s rank correlation coefficient
